# The Synergistic Effect of Polysaccharides and Silane Coupling Agents on the Properties of Calcium Phosphate-Based Bone Substitutes

**DOI:** 10.3390/ijms26188910

**Published:** 2025-09-12

**Authors:** Piotr Pańtak, Joanna P. Czechowska, Vladyslav Vivcharenko, Annett Dorner-Reisel, Aneta Zima

**Affiliations:** 1 Faculty of Materials Science and Ceramics, AGH University of Krakow, al. A. Mickiewicza 30, 30-059 Krakow, Poland; 2 Department of Tissue Engineering and Regenerative Medicine, Faculty of Medical Sciences, Medical University of Lublin, Chodzki 1, 20-093 Lublin, Poland; 3 Faculty of Mechanical Engineering, Schmalkalden University of Applied Sciences, 98574 Schmalkalden, Germany

**Keywords:** calcium phosphates, bioceramics, polysaccharides, hybrid materials, silane coupling agents

## Abstract

In this study, novel hybrid cementitious materials composed of calcium phosphates and polysaccharides were obtained and developed. Moreover, the impact of two distinct silane coupling agents—tetraethyl orthosilicate (TEOS) and 3-glycidoxypropyltrimethoxysilane (GPTMS)—on the physicochemical and biological properties of the resulting materials was systematically analyzed. Comprehensive assessments were conducted to evaluate the chemical and phase compositions (using XRF, XRD, FTIR), setting behavior, mechanical strength, microstructure (SEM), porosity, in vitro chemical stability, and biological performance of bone cements. Notably, the synergistic effect of polysaccharides and silane coupling agents significantly enhanced the compressive strength of the cements, increasing it to 19.34 MPa. Additionally, the integration of citrus pectin into the liquid phase, along with the inclusion of hybrid hydroxyapatite–chitosan granules, not only enabled the formation of materials with high surgical handiness but also improved the materials’ physicochemical characteristics. The findings from this study emphasize the beneficial role of silane coupling agents in improving the properties of calcium phosphate-based bone substitutes. The developed materials demonstrate substantial potential for use in bone tissue engineering according to ISO 10993. However, further in vitro and in vivo studies are required to confirm their safety and effectiveness.

## 1. Introduction

Calcium phosphate cements (CPCs) based on α-tricalcium phosphate (α-TCP) are widely used in contemporary regenerative medicine, particularly in the management of bone defects [1]. A key advantage of CPCs lies in their inherent bioactivity, which facilitates direct interaction with bone tissue and promotes regenerative processes of bone healing. The superior biological performance of calcium phosphate cements is intrinsically linked to their chemical composition, which closely resembles that of natural bone mineral. The bioactivity of CPCs is primarily attributed to their capacity to form a hydroxyapatite layer on their surface upon contact with physiological fluids, thereby fostering integration with the surrounding bone matrix [2]. Additionally, their properties promote the growth of new bone tissue at the site of application, which makes CPCs highly suitable for various clinical applications, including defect repair, bone stabilization, and controlled drug delivery [3,4,5].

Despite their outstanding biological properties, calcium phosphate bone cements (CPCs) exhibit certain limitations that hinder their widespread application in orthopedic and dental surgery [6]. The biggest disadvantage of CPCs is their low mechanical strength and pronounced brittleness [7]. These mechanical deficiencies are particularly troublesome in load-bearing regions or areas exposed to significant and dynamic mechanical forces, thereby limiting CPCs’ efficacy and durability in such environments [8]. To overcome these limitations, research on CPC modification focuses on improving their mechanical properties while maintaining their bioactivity. One of the most promising approaches is the introduction of polymers, whether synthetic or natural, into the composition of calcium phosphate cements [9,10]. For example, Czechowska et al. [11] investigated the influence of one of the polysaccharides, namely sodium alginate and methylcellulose, on the physiochemical properties and in vitro behavior of α-TCP-based cementitious materials and confirmed the beneficial influence of polymeric addition on their compressive strength. Watanabe et al. [12] examined α-TCP-based bone cements modified with polyvinyl alcohol. They determined that higher concentrations of polyvinyl alcohol significantly enhanced the mechanical properties of the developed materials. Dziadek et al. [13], through the addition of another example of polysaccharides, low-esterified pectin, developed novel bone substitutes with high surgical handiness and improved mechanical properties. Polymers also enhance the flexibility and mechanical resistance of CPCs, making them more suitable for dynamically loaded areas. However, they may accelerate material degradation [14]. Another approach to strengthening CPCs involves adding fillers, such as fibers or granules, which improve mechanical properties and modify stress distribution within the material [15,16,17]. A particularly interesting material solution involving bone substitute materials based on calcium phosphates containing aggregates in the form of granules is biomicroconcrete, where the granules acting as aggregates enhance the resistance to the brittle fracture of these materials, like the behavior observed in technical concrete applications [18].

Although the use of polymers and aggregates significantly improves the mechanical properties of CPCs, these composites still have limitations, prompting the ongoing search for new solutions. One of the latest and most promising approaches is the use of silane coupling agents (SCAs) as cement modifiers [19]. SCAs are chemical compounds capable of reacting with both organic and inorganic components of materials, which influences both physicochemical and biological properties of composites [20]. For example, Kouhi et al. [21] modified bredigite (BR) nanoparticles with 3-glycidoxypropyltrimethoxysilane (GPTMS) to improve their dispersibility in a polyhydroxybutyrate-co-hydroxyvalerate (PHBV) matrix. They demonstrated that the PHBV scaffolds containing GPTMS-modified bredigite (G-BR) exhibited enhanced mechanical properties and better nanoparticle dispersion. Furthermore, materials demonstrated improved cell attachment and proliferation, which makes them promising candidates for bone tissue engineering. On the other hand, Bravaya et al. [22] modified alumina nanofibers with various silane agents to create hybrid ethylene–propylene copolymer materials. In this study, trialkoxysilanes with alkenyl and alkyl functional groups were used for the modification of silane coupling agents, improving the mechanical properties of the final materials and nanofiller dispersion. Varghese et al. [23] optimized the surface treatment of glass fibers in dental composites using three different silane coupling agents (3-methacryloxypropyltrimethoxysilane (3-MPS), 3-glycidoxipropyltrimethoxysilane (3-GPS), or 8-methacryloxyoctyltrimethoxysilane (8-MOTS)), incorporating the treated fibers into a dental resin. They found that the presence of silane in certain concentrations (1.4 wt.% for 8_MOTS and 0.8 wt.% for 3-MPS) significantly improved the overall mechanical performance of the composites.

Thus, although silane coupling agents are not yet widely used in bone substitute materials, the scientific literature suggests their potential benefits. It is believed that their incorporation can significantly enhance the mechanical properties of cementitious materials, primarily by improving the bonding between the individual components. These features make SCAs promising candidates for further research and development in biomaterial engineering.

The objective of this study was to develop, obtain, and evaluate novel hybrid bone substitute materials based on highly reactive, self-setting α-TCP (α-tricalcium phosphate) powder combined with hybrid hydroxyapatite–chitosan granules. The liquid phase consisted of a blend of citrus pectin gel and disodium phosphate. Additionally, this study aimed to investigate how the modification of α-TCP with two different silane coupling agents, specifically tetraethyl orthosilicate (TEOS) and 3-glycidoxypropyltrimethoxysilane (GPTMS), influences their physicochemical and biological properties.

## 2. Results and Discussion

### 2.1. Chemical and Phase Composition

The X-ray fluorescence analysis confirmed the presence of silicon in all modified powders, with a baseline level of 0.029 ± 0.002 wt.% in the non-modified powder. As anticipated, the silicon content increased with the addition of silane coupling agents (SCAs), reaching 0.223 ± 0.006 wt.% and 0.286 ± 0.004 wt.% for powders modified with 5 wt.% TEOS and GPTMS, respectively. The presence of silicon may enhance the biological properties of the final material, such as osteoconductivity [24,25]. This modification facilitated the enrichment of the powder with silicon ions following α-TCP synthesis, whereas most of the scientific literature focuses on introducing silicon ions during the synthesis of calcium phosphate-based materials [26,27].

**Figure 1:**
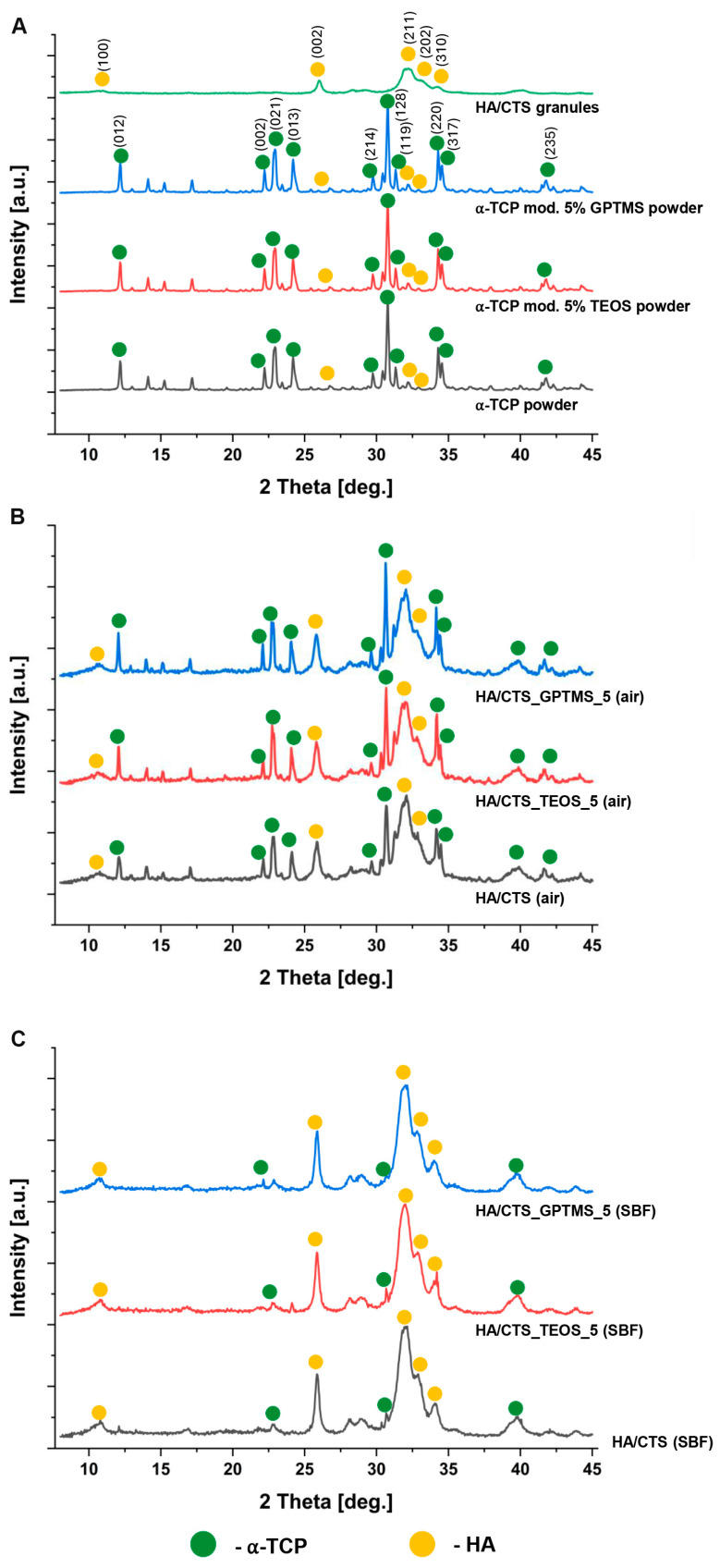
X-ray diffraction (XRD) patterns: initial powders and granules (**A**), cementitious materials after 7 days of setting and hardening in air (**B**), and incubation in SBF for 7 days (**C**).

The XRD analysis showed that both the initial α-TCP and SCA-modified α-TCP powders were primarily composed of α-TCP (97–98 wt.%), with a minor presence of hydroxyapatite (2–3 wt.%). In contrast, the hybrid HA-CTS powder contained only one crystalline phase, hydroxyapatite (Figure 1A). The diffractograms of the cementitious materials, after setting and hardening in air for 7 days and after 7 days of incubation in SBF, revealed the presence of two crystalline phases: hydroxyapatite and a small amount of α-TCP (Figure 1B,C). No additional silicon-containing crystalline phases were identified by XRD.

The quantitative analysis of the diffractograms indicated that in a humid environment, the α-TCP phase displayed thermodynamic metastability and underwent near-complete hydrolysis, leading to the formation of calcium-deficient hydroxyapatite (CDHA) (Table 1). This transformation was previously observed inter alia by E. Şahin [28] and L. Yubao [29]. The process of hydrolysis is believed to affect the material’s properties and performance in biological applications.

**Figure 2:**
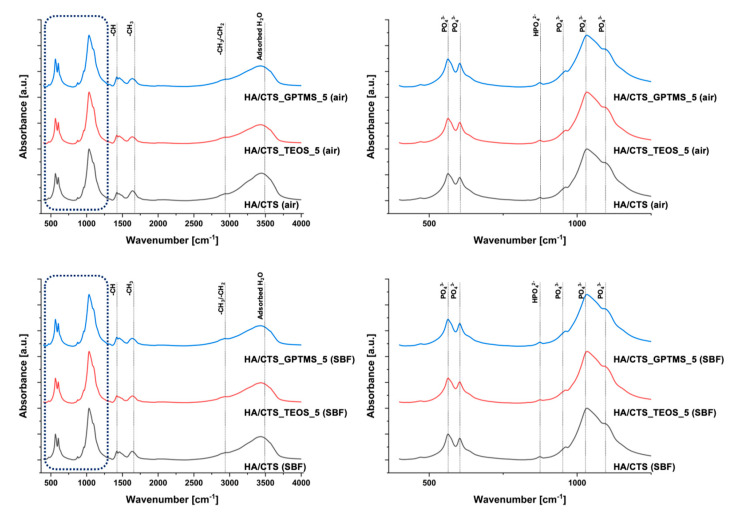
FT-IR spectra of investigated materials.

The FTIR study results support the XRD findings and confirm the presence of functional groups characteristic of calcium phosphates and polymers. The infrared spectra of the developed materials, after setting and hardening in air for 7 days, as well as after incubation in simulated body fluid (SBF), are shown in Figure 2.

The FTIR spectra of the materials revealed distinct bands at approximately 600 and 560 cm^−1^, corresponding to the bending vibrations of the PO_4_^3−^ groups, and around 965 and 1020 cm^−1^, associated with the stretching vibrations of these groups. A broad band in the range of approximately 3000–3800 cm^−1^ was attributed to absorbed water. Additionally, an absorption band at around 870 cm^−1^ was linked to the HPO_4_^2−^ groups, indicating the presence of non-stoichiometric hydroxyapatite. In the same spectral range (approximately 873–875 cm^−1^), there were also possible carbonate bonds in the material. The band at 1424 cm^−1^ suggested partial substitution of CO_3_^2−^ within the hydroxyapatite structure. FTIR analysis also confirmed the presence of chitosan and amidated citrus pectin, with an absorption band at approximately 2930 cm^−1^ corresponding to alkyl C-H stretching vibrations and the band around 1650 cm^−1^ attributed to N-H bending vibrations of primary amines. The presence of these bands suggests the formation of electrostatic and/or hydrogen bonds, likely forming polyelectrolyte complexes at the interface between the hybrid powder and pectin within the cements. The low concentration of silane coupling agents in the pastes, combined with overlapping bands from phosphates, may account for the absence of visible peaks corresponding to Si–O–Si, Si-OH, Si–C, and C–H bonds associated with the silane coupling agents [30,31,32].

### 2.2. Setting Times

The setting process of self-setting calcium phosphate-based cementitious pastes begins upon mixing the powder and liquid phases, resulting in a viscous paste whose rheological properties evolve until the material fully hardens. The setting times of the bone cements ranged from 5.5 to 12.0 min for the initial setting time and 11.5 to 19.5 min for the final setting time (Table 2).

The solid phase of the developed pastes consisted of highly reactive α-tricalcium phosphate powder. When this powder comes into contact with the water, it hydrolyzes to calcium-deficient hydroxyapatite, as described by the following Equation (1) [33,34]:

3Ca_3_(PO_4_)_2_ + H_2_O → Ca_9_(PO_4_)_5_(HPO_4_)OH (1)

During hydrolysis, the paste undergoes a setting and hardening process, which involves the nucleation and growth of CDHA crystals. Initially, the powder particles within the paste undergo restructuring, transforming the fluid paste into a rigid, yet weak, monolith. Subsequently, the strength of the material increases as the hardening process continues.

The setting process of cementitious materials based on α-TCP is influenced by many factors. Primarily, it is dependent on the amount of highly reactive α-TCP powder in the materials’ formulation. Additional factors, such as the presence of setting accelerators in the liquid phase or polymers in the mixture, also play significant roles. In the developed materials, disodium hydrogen phosphate was used to accelerate setting and counteract the elongation of the setting process caused by citrus pectin [35]. Since the same liquid phase was used for all formulations, variations in setting times were attributed to differences in the solid phase composition, especially the incorporation of silane coupling agents as modifiers of α-TCP powders. It appears that the presence of SCAs slightly reduced the setting time of the cement pastes. This effect may be attributed to the hydrolysis of silane coupling agents upon exposure to water, leading to their condensation and potentially contributing to a minor acceleration in the setting process. This phenomenon is well-described in previous studies [27,36]. Another possible explanation involves the SCAs acting as sources of silicon ions, which are known to enhance the solubility of α-TCP and accelerate its setting compared to unmodified powders.

### 2.3. Mechanical Strength

**Figure 3:**
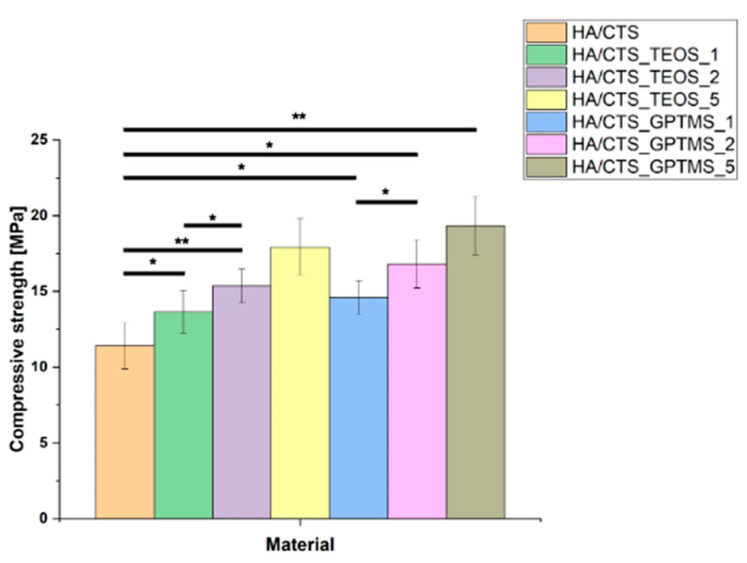
Compressive strength of developed bone substitutes. Statistical significance is indicated as *p* < 0.05 (*) and *p* < 0.01 (**).

The compressive strength of the developed biomaterials ranged from 11.42 ± 1.12 MPa to 19.34 ± 0.94 MPa. It was observed that the compressive strength of the materials depended on the amount of coupling agents used. An increase in the quantity of the modifier resulted in higher mechanical strength, regardless of the type of coupling agent employed. The inclusion of silane coupling agents (SCAs) enhanced the strength of the bone cements, with the highest value recorded for the material HA/CTS_GPTMS_5, which contained 5 wt.% of GPTMS (Figure 3). Statistical analysis using one-way ANOVA and Tukey’s HSD post hoc tests revealed that the differences between the control and modified materials were significant.

Silane coupling agents used as modifiers for α-TCP can synergistically enhance the mechanical properties of the resulting bone cements by forming additional bonds within the material structure and causing better adhesion between the material’s compounds. Moreover, the use of natural polymers in the composition of the developed cementitious materials further reinforced this effect due to synergistic interactions between the functional groups of the polymers and those originating from the coupling agents.

The improvement in mechanical strength likely arises from chemical interactions between the components, leading to the creation of Si-O-Si, Si-O, or Si-O-P bonds, which result in hybrid-type materials. A similar positive effect of silane coupling agents on the mechanical performance of various biomaterials has also been reported in previous studies. For example, Ji et al. [37] developed hydroxyapatite-based scaffolds by modifying the surface of HA pellets with silane coupling agents containing methacrylate, amine, and carboxylic acid functional groups to enhance their mechanical properties for dental and bone regeneration applications. They found that coating with carboxylic acid-functionalized silane significantly improved the mechanical strength and biocompatibility of the scaffolds, making them suitable as bone filler composites in tissue engineering. Vaz et al. [38] showed beneficial application of different coupling agents to obtain starch/ethylene-vinyl alcohol copolymer/hydroxyapatite composites characterized by increased mechanical resistance due to improved adhesion between material components. On the other hand, Kotha et al. [39] evaluated the impact of using a silane coupling agent (methacryloxypropyl-trichlorosilane) on the mechanical properties of steel fiber-reinforced acrylic bone cements. They studied the tensile and fracture properties of cements reinforced with silane-coated or uncoated 316 L stainless steel fibers and found that the interfacial shear strength and mechanical properties were significantly improved with silane-coated fibers. Furthermore, the improvement in the mechanical strength of the developed bone cement materials was achieved due to interactions between the chitosan present in the hybrid granules and the citrus pectin incorporated in the liquid phase, as well as the functional groups derived from the silane coupling agents. A similar effect was previously observed by Pańtak et al. [40]. The authors developed and evaluated hybrid bone scaffolds fabricated using the robocasting technique. In their study, it was demonstrated that the use of silane coupling agents and citrus pectin enhanced the mechanical strength of the biomaterials through interactions between calcium phosphates, polymers, and the silane coupling agents.

### 2.4. Porosity

In the developed materials, the incorporation of silane coupling agents resulted in a noticeable reduction in porosity. The HA/CTS biomaterial without SCA modification exhibited a porosity of 58.3 ± 0.5 vol.%, while materials modified with TEOS and GPTMS showed significantly lower porosity values of 47.6 ± 0.5 vol.% and 46.4 ± 0.5 vol.%, respectively. The observed decrease in porosity closely correlated with the compressive strength results. Specifically, the reduction in porosity in materials containing silane coupling agents contributed to an improvement in their mechanical strength.

**Figure 4:**
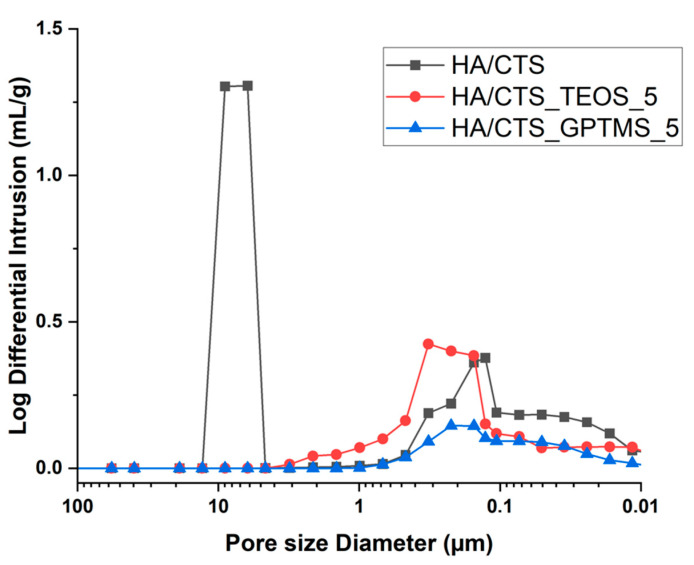
Distribution and size of pores in the obtained bone substitutes.

Additionally, the pore size distribution plots revealed distinct differences: the unmodified HA/CTS cement displayed a bimodal porosity distribution with larger pores, approximately 10 microns in size, while no such large pores were observed in the SCA-modified materials (Figure 4).

The observed decrease in porosity and the absence of large pores in the modified materials can be attributed to the use of SCAs, which are known to enhance adhesion between different components of a material [41]. In this case, the application of SCAs likely improved the adhesion of the α-TCP powder to the hybrid granules, resulting in a more compact structure with fewer and smaller pores. Silane coupling agents are commonly used to promote interfacial bonding in composite materials, thereby improving mechanical properties and reducing porosity, as reported in previous studies [42,43].

### 2.5. Microstructure

**Figure 5:**
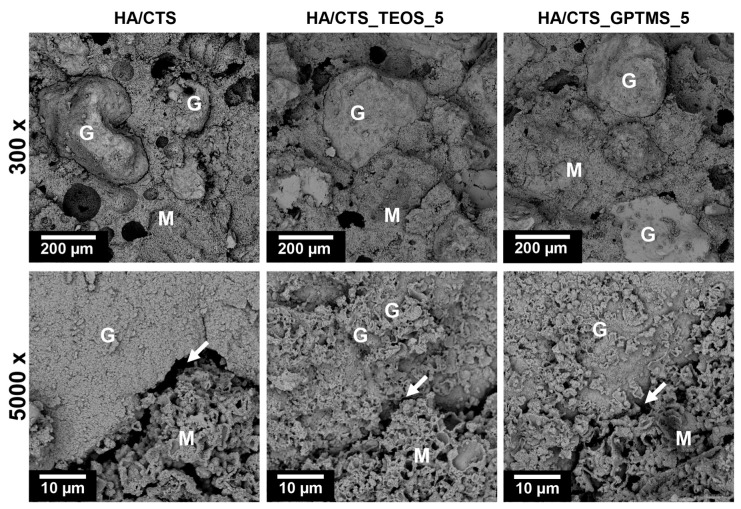
Microstructure of developed bone cements (M—cementitious matrix; G—hybrid HA/CTS granules, arrows—matrix/granule interface).

The set and hardened materials displayed a uniform microstructure, consisting of hybrid granules embedded in a cementitious matrix with both macro- and micropores. The hybrid granules were visible and evenly distributed in the material (Figure 5). Interestingly, the use of SCAs improved the adhesion between the cementitious matrix and hybrid granules (highlighted with arrows). Similar microstructures in α-TCP-based biomaterials intended for bone tissue replacement have been reported in other studies [35].

### 2.6. Chemical Stability and Bioactivity In Vitro

**Figure 6:**
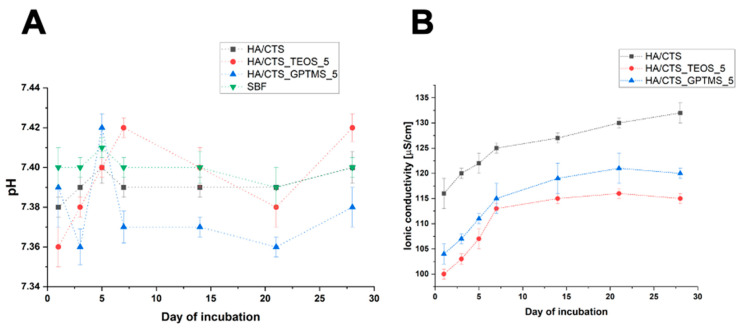
pH (**A**) and ionic conductivity (**B**) vs. time of incubation of developed biomaterials.

Evaluating the chemical stability of candidate materials for bone substitution is crucial, as it helps predict how the material will behave after the implantation procedure. Figure 6 illustrates the pH changes in the SBF and the ionic conductivity variations in distilled water during the immersion of the samples.

The pH levels of the SBF surrounding the incubated samples remain near the physiological values, fluctuating between 7.36 and 7.42. The introduction of TEOS and GPTMS only had a minor effect on the pH of the solution. Comparable pH levels for incubated calcium phosphate-based bone substitutes have been reported in other studies [11].

The ionic conductivity during the samples’ incubation in distilled water also depended on their composition (Figure 6B). The ionic conductivity of the distilled water around the incubated HA/CTS was the highest and ranged from approximately 140 to 181 μS/cm. In the case of SCA-modified materials, these values were slightly lower: between 100 and 116 μS/cm for HA/CTS_TEOS_5 and 116 and 133 μS/cm for HA/CTS_GPTMS_5. The observed phenomenon could be attributed to the faster degradation and hydrolysis of HA/CTS cement agents in aqueous solutions [44]. The ionic conductivity of all tested biomaterials was comparable to previously studied chemically bonded biomaterials based on α-TCP.

**Figure 7:**
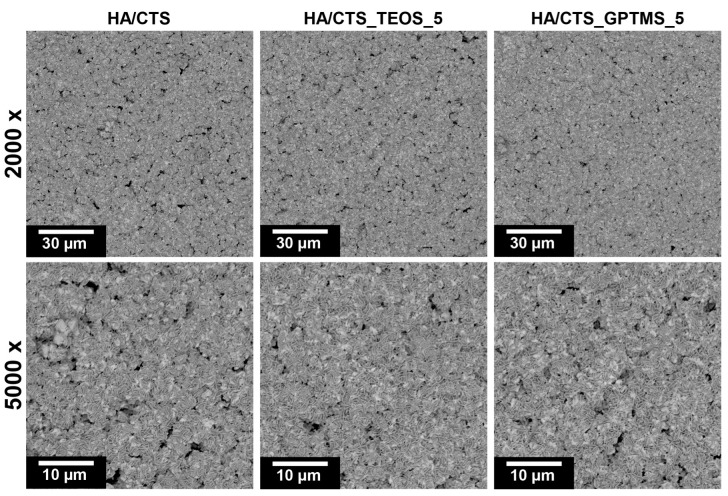
Microstructure of materials’ surfaces after 7 days of incubation in SBF.

After 7 days of incubation in simulated body fluid (SBF), all prepared materials were fully covered by a plate-like apatitic structure, which, according to Kokubo and Takadama’s criteria, confirms in vitro bioactive potential of composites (Figure 7) [45].

### 2.7. Cytotoxicity Tests

**Figure 8:**
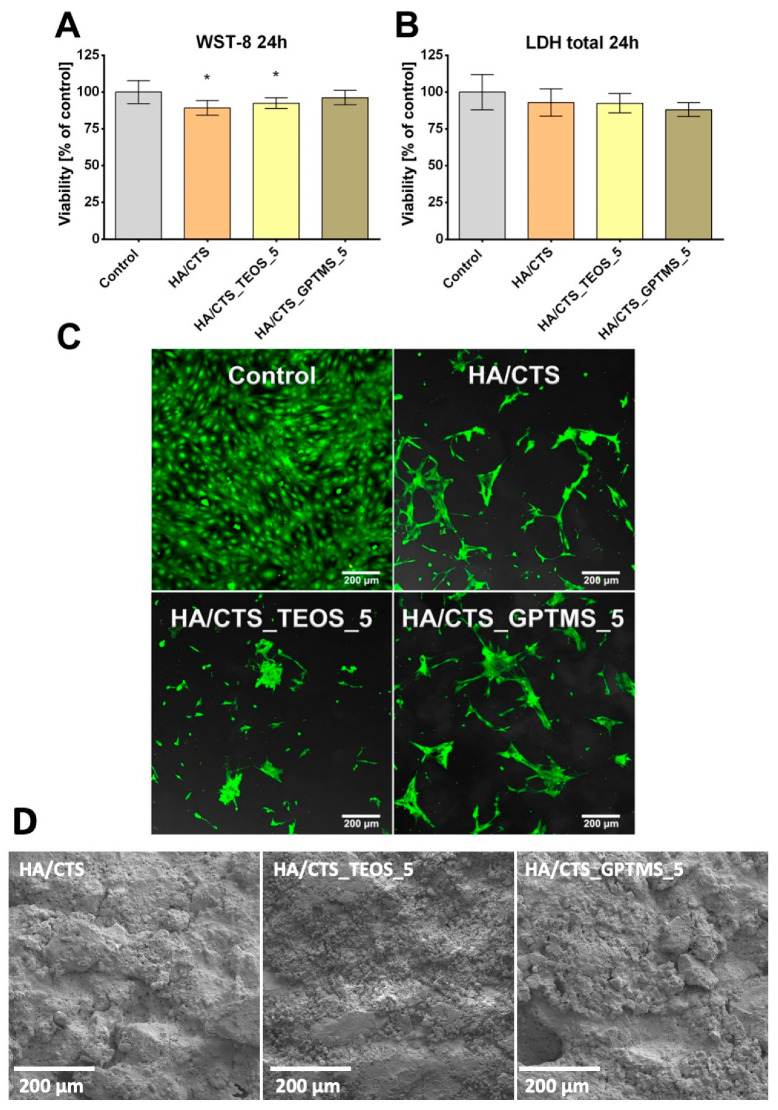
Cytocompatibility evaluation of the tested cementitious samples against mouse calvarial preosteoblast cell line: (**A**) WST-8 test conducted using scaffold extracts (* statistically significant results compared to the control, *p* < 0.05, one-way ANOVA followed by Tukey’s test); (**B**) LDH total test conducted using scaffold extracts; (**C**) direct contact cytotoxicity assay performed with a Live/Dead staining kit (green fluorescence indicating viable cells, red fluorescence indicating dead cells); (**D**) SEM of developed materials’ surfaces.

The cytotoxicity of the tested cementitious samples was evaluated based on the metabolic activity of MC3T3-E1 cells after exposure to biomaterial extracts, using the WST-8 test. Cell viability in all tested samples was high, exceeding 90%, which indicates a lack of cytotoxicity, as defined by ISO 10993-5:2009 (Figure 8A).

HA/CTS and HA/CTS_TEOS_5 samples exhibited slightly lower viability values in the WST-8 assay compared to the control, which can be attributed to the negative effect of biomaterial extracts on mitochondrial dehydrogenase activity measured during the experiment. The absence of cytotoxicity was further confirmed by the LDH total assay performed after cell lysis, where the measured LDH activity correlated with the cell number in the population. The conducted test revealed no statistically significant differences between the scaffolds and the negative control, confirming the non-cytotoxic character of the samples (Figure 8B). The cytotoxicity of the tested samples was further evaluated using a direct contact test. Mouse preosteoblasts were cultured on the scaffolds for 2 days and then visualized through fluorescent staining (Figure 8C). The distribution of the observed calvarial preosteoblasts reflects the microstructure of the surface of the cementitious materials. It can be noted that the cells spread out within the depressions present on the surface of the analyzed materials (Figure 8D).

## 3. Materials and Methods

### 3.1. Materials

#### 3.1.1. Synthesis of α-TCP and SCA-Modified α-TCP Powders

The initial α-TCP powder was synthesized via a wet chemical method following a previously described procedure [46,47]. Ca(OH)_2_ (≥99.5%, POCH, Gliwice, Poland) and H_3_PO_4_ (85.0%, POCH, Gliwice, Poland) at the Ca/P molar ratio of 1.5 were applied as reagents. After aging and drying, the precipitate underwent a calcination at 1250 °C for 5 h, grinding in an attritor mill for 4 h, and sieving below 63 µm. To modify the surface of α-TCP powder, the 1, 2, and 5 wt.% solutions of TEOS (T) or GPTMS (G) (≥99.5%, Sigma-Aldrich, St. Louis, MO, USA) in ethanol (99.8 wt.%, POCH, Gliwice, Poland) were applied according to Pańtak et al. [19]. The anhydride solvent was used to avoid the hydrolysis of both α-TCP and silane coupling agents. α-TCP powder was added to the SCA solution at the liquid-to-powder (L/P) ratio of 0.25 and stirred in a magnetic stirrer for 4 h. The sedimented powder was then aged for 1 h and silanized at 115 °C for 4 h. Prior to preparing the samples, the powders were sieved below 63 µm.

#### 3.1.2. Synthesis of Hybrid Hydroxyapatite/Chitosan Granules

Hybrid HA/chitosan (CTS) granules, containing 17 wt.% CTS, were synthesized using a modified wet chemical method, following the procedure outlined previously by Zima [48]. Briefly, phosphoric acid (85.0%, POCH, Gliwice, Poland) was directly added to a 10 wt.% CTS solution in 0.5 wt.% acetic acid (98.0%, POCH, Gliwice, Poland). This mixture was then carefully dripped into a suspension of Ca(OH)_2_ (Merck, Darmstadt, Germany) for HA precipitation. The molar ratio of Ca/P during the synthesis was within the range of 1.65–1.67. The CTS used was of medium molecular weight (~100,000 kDa) with a deacetylation degree of ≥75.0% and a viscosity ranging from 200 to 800 CPS (Sigma-Aldrich, St. Louis, MO, USA). After aging the suspension for 24 h, it was decanted. The HA/CTS precipitate was washed with distilled water, centrifuged, and dried. Prior to preparing the samples, the granules were ground and sieved. For the preparation of the samples, granules of sizes 300–400 µm were used.

### 3.2. Preparation of Bone Cements

Developed biomaterials are composed of solid phase (setting powder and aggregate) and liquid phase, and when mixed, they create a paste that sets in situ. In this study, seven types of powder batches containing α-TCP, modified α-TCP, and HA/CTS granules were used as the solid phase (Table 3). The liquid-to-powder (L/P) ratio was selected based on preliminary studies, and it was 0.5 g/g.

### 3.3. Methods

#### 3.3.1. Chemical and Phase Composition

The chemical composition of the initial powders was examined using X-ray fluorescence (XRF) with the WDXRF Axios Max (PANalytical, Malvern, UK). X-ray diffraction (XRD) was used to identify crystalline phases, employing Cu Kα radiation (1.54 Å) at 30 kV and 10 mA. The analysis covered a 2θ range of 5–45° with 0.04 intervals, scanning at a speed of 2.5°/min, using a D2 Phaser diffractometer (Bruker, Ballirica, MA, USA). Diffractograms were compared with the International Centre for Diffraction Data for α-TCP (00-009-0348) and hydroxyapatite (HA; 01-076-0694). Phase quantification was performed using TOPAS software (Bruker, Billerica, MA, USA) based on Rietveld refinement. All measurements were performed in triplicate, with results presented as mean ± SD.

The structural analysis of the obtained biomaterials after setting and hardening was carried out using Fourier Transform Infrared (FTIR) spectroscopy, covering a scanning range of 400–4000 cm^−1^ with a resolution of 4 cm^−1^, using a BioRad FTS 6000 spectrometer (Vertex 70&70v, Bruker, Billerica, MA, USA). The positions of the FTIR bands were determined based on the center of weight. Baseline correction, normalization, and spectral analysis were conducted using Spectragryph software (v1.2.15, Friedrich Menges, Oberstdorf, Germany).

#### 3.3.2. Setting Times

The setting times were determined according to the ASTM C266-20 standard using Gilmore Needles (Humbold MFG Co., Norridge, IL, USA) [49]. The apparatus included two steel-weighted needles: the initial setting needle, weighing 113 g with a diameter of 2.12 mm, and the final setting needle, weighing 453.6 g with a diameter of 1.06 mm. Cement pastes were placed in a form measuring 8 mm × 10 mm × 5 mm, and the needle was gently applied to the surface. The setting time was recorded when the needle no longer left a complete circular mark. All tests were conducted at 22 ± 1 °C, with results averaged over three measurements, including standard deviations (SDs).

#### 3.3.3. Mechanical Strength

For mechanical testing, cylindrical samples with dimensions of 12 mm in height and 6 mm in diameter were fabricated using Teflon molds. The samples were extracted from the molds between their initial and final setting times and allowed to cure in air for 7 days. Following this curing period, compressive strength was evaluated using a universal testing machine (Instron 3345, Instron, Norwood, MA, USA). The cementitious samples were subjected to uniaxial compression at a crosshead speed of 1.0 mm/min. The compressive strength values were presented as the averages of 15 measurements, along with their standard deviations (SDs).

#### 3.3.4. Porosity

The open porosity and pore size distribution of the foamed cements were evaluated using mercury intrusion porosimetry (MIP) with the AutoPore IV porosimeter (Micrometrics, Norcross, GA, USA). Dried sample fragments were placed in the penetrometer, which was then positioned in the low-pressure chamber of the apparatus and degassed. Mercury was introduced into the penetrometer, and the volume of mercury intrusion was recorded as the pressure increased. After completing the low-pressure measurements, the penetrometer was moved to the high-pressure chamber for additional testing. All measurements were performed in triplicate.

#### 3.3.5. Microstructure

To observe the microstructures of the fractured samples and assess the bioactive potential of the materials, a PhenomPure scanning electron microscope (SEM) from Thermo Fisher Scientific (Waltham, MA, USA) was employed. Before examination, the samples were coated with a thin layer of gold using a low deposition rate to prevent charge accumulation and enhance image resolution.

#### 3.3.6. Chemical Stability and Bioactivity

To assess the in vitro chemical stability of the materials, cylindrical samples with dimensions of 3 mm in height and 6 mm in diameter were placed in plastic containers with 20 mL of either simulated body fluid (SBF) or distilled water and incubated at 37 °C for 4 weeks. During this period, the ionic conductivity and pH of the surrounding solutions were monitored using a Seven Compact Duo pH/conductometer (Mettler Toledo, Columbus, OH, USA). The in vitro bioactivity of the bone substitutes was evaluated through scanning electron microscopy (SEM), focusing on the formation of apatite layers on the material surfaces after 7 days of incubation in SBF.

#### 3.3.7. Cell Studies

In vitro cytocompatibility studies were performed using a mouse primary calvarial preosteoblast cell line (MC3T3-E1 Subclone 4, CRL-2593, ATCC-LGC standards, Teddington, UK). MC3T3-E1 cells were cultured in Alpha Minimum Essential Medium (GIBCO, Life Technologies, Carlsbad, CA, USA) supplemented with 10% fetal bovine serum (FBS, Pan-Biotech GmbH, Aidenbach, Bavaria, Germany) and antibiotics (100 U/mL of penicillin and 0.1 mg/mL of streptomycin) (Sigma-Aldrich Chemicals, Warsaw, Poland). The cells were maintained at 37 °C with 5% CO_2_ in an air atmosphere.

Before the cell culture experiments, all samples were sterilized by ethylene oxide. The cytotoxicity of the fabricated cementitious samples was evaluated based on MC3T3-E1 viability after the cells were exposed to the liquid biomaterial extracts in accordance with ISO 10993-5 (2009). First, mouse preosteoblasts at a concentration of 1.5 × 10^5^ cells/mL were seeded in flat-bottom 96-multiwell plates and cultured for 24 h. Next, the culture medium was replaced with sample extracts prepared according to ISO 10993-12:2021. The cell culture medium incubated in a polystyrene plate served well as a negative control. After 24 h, the cytotoxicity of the bone cement samples was assessed using WST-8 and LDH total assays, according to the manufacturers’ instructions (Sigma-Aldrich Chemicals, Warsaw, Poland). The cytotoxicity of the tested samples was also estimated using a Live/Dead Double Staining Kit (Sigma-Aldrich Chemicals, St. Louis, MO, USA). The study was conducted in direct contact of mouse preosteoblasts with biomaterials. First, cells were seeded directly on the pre-soaked samples at a concentration of 1×10^5^ and cultured for 48 h. Next, the cells were fluorescently stained using a Live/Dead Double Staining Kit according to the manufacturer’s instructions and visualized using a confocal laser scanning microscope (CLSM, Olympus Fluoview equipped with FV1000, Olympus Corporation, Tokyo, Japan). Cells seeded on the polystyrene surface were used as a control.

#### 3.3.8. Statistics

The statistical analysis of the obtained results was performed using a one-way analysis of variance (ANOVA) with a post hoc Tukey Honestly Significant Difference (HSD) test for comparing multiple treatments. Statistically significant differences were indicated by * (*p* < 0.05) and ** (*p* < 0.01). In the case of in vitro cell culture tests, statistical significance was considered at *p* < 0.05.

## 4. Conclusions

In this research, innovative hybrid bone substitutes were developed and investigated. The cementitious materials produced consisted of highly reactive α-TCP powder (either unmodified or modified with silane coupling agents), hybrid hydroxyapatite–chitosan granules, and citrus pectin used with disodium phosphate as the liquid phase. This study focused on the impact of two distinct coupling agents—tetraethyl orthosilicate (TEOS) and 3-glycidoxypropyltrimethoxysilane (GPTMS)—on the physicochemical and biological properties of the bone substitutes. The unique characteristics of these materials stem from the hybrid system, which synergistically relies on chemical interactions between the hybrid granules, pectin, and SCA-modified tricalcium phosphates. These chemical interactions contribute to reducing porosity and strengthening the bone substitutes, resulting in an increase in compressive strength from approximately 11.42 to 19.34 MPa. Notably, the silane coupling agents had beneficial effects on the materials’ microstructure, chemical stability, or biological performance. All the developed cements exhibited bioactive potential in vitro, indicating their suitability for further biological research. Additionally, the use of easily functionalizable polymers like chitosan and citrus pectin, alongside silane coupling agents, allows for future modifications of the materials with drugs or other bioactive compounds. This research highlights the favorable properties of the bone substitutes, laying the groundwork for future in vitro and in vivo investigations.

## Figures and Tables

**Table 1:** Phase composition of the obtained materials after 7 days of setting and hardening in air or after 7 days of incubation in SBF.

Material	7 Days in Air		7 Days in SBF	
	*α-TCP,* *wt.%*	*Hydroxyapatite, wt.%*	*α-TCP,* *wt.%*	*Hydroxyapatite, wt.%*
HA/CTS	58 ± 2	42 ± 2	8 ± 2	92 ± 2
HA/CTS_TEOS_5	52 ± 1	48 ± 1	7 ± 1	93 ± 1
HA/CTS_GPTMS_5	51 ± 2	49 ± 2	6 ± 1	94 ± 2

**Table 2:** Setting times of developed materials.

Material	Initial Setting Time (ti) [min]	Final Setting Time (tf) [min]
HA/CTS	11.0 ± 1.0	19.5 ± 1.5
HA/CTS_TEOS_1	8.5 ± 0.5	17.5 ± 0.5
HA/CTS_TEOS_2	12.0 ± 1.5	15.0 ± 0.5
HA/CTS_TEOS_5	6.5 ± 1.0	15.0 ± 1.0
HA/CTS_GPTMS_1	10.0 ± 1.5	17.5 ± 1.5
HA/CTS_GPTMS_2	7.5 ± 0.5	14.5 ± 1.0
HA/CTS_GPTMS_5	5.5 ± 0.5	11.5 ± 1.0

**Table 3:** The initial composition of developed materials.

Material	Solid Phase (P)	Liquid Phase (L)	L/P [g/g]
HA/CTS	α-TCP: HA/CTS granules 3:2 by weight	1.0 wt.% Na_2_HPO_4_ solution in 2.5 wt.% citrus pectin gel	0.5
HA/CTS_TEOS_1	α-TCP (1 wt.% TEOS): HA/CTS granules 3:2 by weight		
HA/CTS_TEOS_2	α-TCP (2 wt.% TEOS): HA/CTS granules 3:2 by weight		
HA/CTS_TEOS_5	α-TCP (5 wt.% TEOS): HA/CTS granules 3:2 by weight		
HA/CTS_GPTMS_1	α-TCP (1 wt.% GPTMS): HA/CTS granules 3:2 by weight		
HA/CTS_GPTMS_2	α-TCP (2 wt.% GPTMS): HA/CTS granules 3:2 by weight		
HA/CTS_GPTMS_5	α-TCP (5 wt.% GPTMS): HA/CTS granules 3:2 by weight		

## Data Availability

The data presented in this study are available on request from the corresponding author.
